# The GyrA-box determines the geometry of DNA bound to gyrase and couples DNA binding to the nucleotide cycle

**DOI:** 10.1093/nar/gks852

**Published:** 2012-09-12

**Authors:** Martin A. Lanz, Dagmar Klostermeier

**Affiliations:** Institute for Physical Chemistry, University of Muenster, Corrensstrasse 30, D-48149 Muenster, Germany

## Abstract

DNA gyrase catalyses the adenosine triphosphate-dependent introduction of negative supercoils into DNA. The enzyme binds a DNA-segment at the so-called DNA-gate and cleaves both DNA strands. DNA extending from the DNA-gate is bound at the perimeter of the cylindrical C-terminal domains (CTDs) of the GyrA subunit. The CTDs are believed to contribute to the wrapping of DNA around gyrase in a positive node as a prerequisite for strand passage towards negative supercoiling. A conserved seven amino acid sequence motif in the CTD, the so-called GyrA-box, has been identified as a hallmark feature of gyrases. Mutations of the GyrA-box abolish supercoiling. We show here that the GyrA-box marginally stabilizes the CTDs. Although it does not contribute to DNA binding, it is required for DNA bending and wrapping, and it determines the geometry of the bound DNA. Mutations of the GyrA-box abrogate a DNA-induced conformational change of the gyrase N-gate and uncouple DNA binding and adenosine triphosphate hydrolysis. Our results implicate the GyrA-box in coordinating DNA binding and the nucleotide cycle.

## INTRODUCTION

Bacterial gyrase is a type II topoisomerase that introduces negative supercoils into DNA in an adenosine triphosphate (ATP)-dependent process (1). In the cell, gyrase removes positive supercoils in DNA that occur during replication and transcription, and regulates the supercoiling homeostasis [reviewed in (2)]. In the absence of ATP, gyrase can also catalyse DNA relaxation and decatenation (3,4).

**Figure 1.**
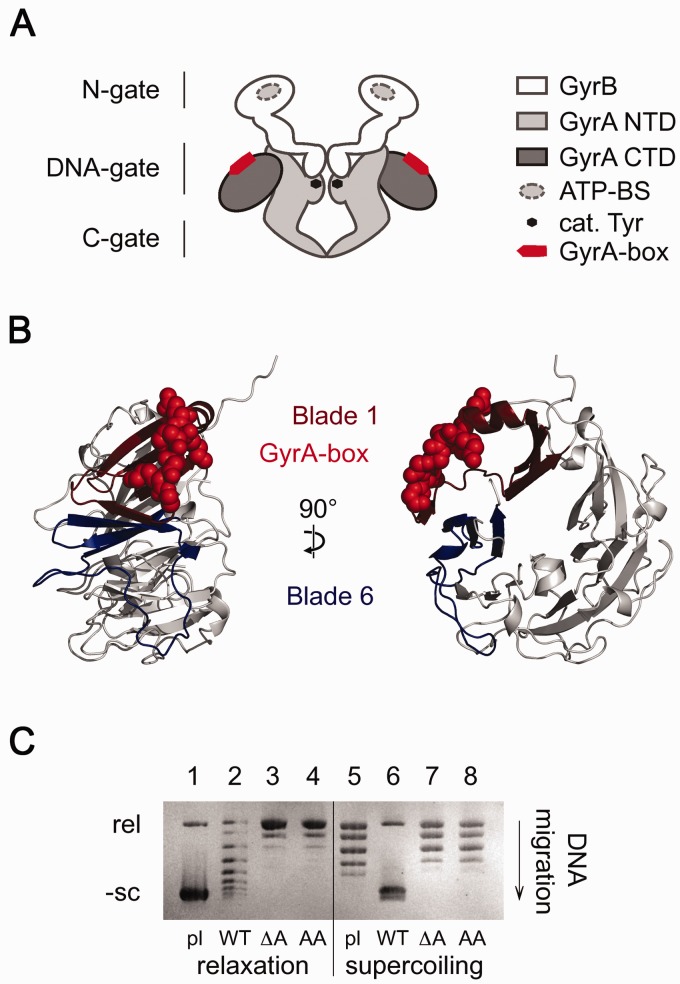
The GyrA-box. (**A**) Cartoon of the GyrB_2_GyrA_2_ heterotetramer. The ATP-binding site (ATP-BS) is located on the GyrB subunits. The catalytic tyrosine residues (cat. Tyr) and the hallmark GyrA-box are found in the NTD and CTD of GyrA. The DNA-gate is formed by the two GyrB and GyrA subunits, and the N- and the C-gate are dimerization interfaces made up by GyrB or GyrA, respectively. (**B**) Side (left) and front views (right) of a homology model of *B. subtilis* GyrA CTDs, using the *X. campestris* GyrA CTD as a template [(5), PDB-entry: 3L6V]. The GyrA-CTD forms a six-bladed β-pinwheel structure. The seven amino acid consensus motif of the GyrA-box, depicted in sphere representation, is located on an extended loop connecting blades 1 and 6 to form a closed ring structure. (**C**) DNA relaxation and supercoiling by gyrase and GyrA-box mutants. Lane 1: Supercoiled plasmid, lanes 2–4: Nucleotide-independent DNA relaxation by wild-type gyrase (WT) and gyrase lacking the GyrA-box (ΔA) and gyrase with the GyrA-box substituted by seven alanine residues (AA) after 120 min incubation, lane 5: Partially relaxed plasmid, lanes 6–8: ATP-dependent DNA supercoiling by wild-type gyrase (WT) and gyrase lacking the GyrA-box (ΔA) and gyrase with GyrA-box substituted by seven alanine residues (AA) after 2 min incubation.

Gyrase is a heterotetrameric enzyme formed by two GyrB and two GyrA subunits (Figure 1A). High-resolution structures of all domains from *Escherichia coli* gyrase have been determined (6–10), but the structure of the complete gyrase is currently unknown. DNA supercoiling is believed to occur by a strand passage mechanism, in which a DNA-segment, the so-called G-segment, is cleaved, and a second DNA-segment, the T-segment, is transported through the gap. Strand passage requires the sequential opening of three protein interfaces, the so-called gates, in a process coupled to ATP hydrolysis (11–13). The central DNA-gate (Figure 1A), formed by the C-terminal domain (CTD) of GyrB and the N-terminal domain (NTD) of GyrA, is the primary DNA binding region that binds, distorts and cleaves the G-segment (14). The CTDs of GyrA contribute to DNA binding by wrapping DNA extending from the DNA-gate around their perimeter (15) and are required for DNA supercoiling (16). The N-gate is formed by the NTDs of GyrB, which dimerize in response to ATP binding (6), leading to N-gate closure in gyrase (17), and it is linked to the capture of the T-segment (18). Interestingly, the N-gate already narrows when DNA is bound to the DNA-gate and wrapped around the CTDs, possibly as a preparation for N-gate closure (17). The C-gate is formed by the GyrA subunits (Figure 1A) and is the last gate to be passed by the transported DNA duplex when it exits from gyrase.

The catalytic core is structurally conserved among type II topoisomerases. In the bacterial type II topoisomerases, gyrase and topoisomerase IV, it is flanked by the CTDs that adopt well-defined structures. The CTDs of *E. coli, Xanthomonas campestris* and *Mycobacterium tuberculosis* gyrase form a highly conserved β-pinwheel structure composed of six blades that assemble into a spiral shape (5,8,19). In contrast, the *Borrelia burgdorferi* gyrase CTD adopts a flat or cylindrical structure, with a more compact interface between blades 1 and 6 than in the spiral structures (20). The GyrA CTD binds DNA of >35 bp (15). The surface charge distribution of the CTD suggests DNA binding sites at the perimeter of the domain, formed by positively charged loops emerging from blades 1, 6, 5 and 4 (20,21). Fluorescence resonance energy transfer (FRET) (20), transient electric dichroism (22) and atomic force microscopy studies (23) have demonstrated that DNA bound to the gyrase CTDs is bent. Deletion of the CTDs converts gyrase into a topoisomerase II that does not supercoil DNA, but catalyses ATP-dependent DNA relaxation (16). Gyrase preferentially binds positively supercoiled DNA, an effect that has been linked to the introduction of positive writhe into DNA by the GyrA CTDs (16,24–26). Writhe introduction has been observed for the spirally shaped GyrA CTDs from *E. coli* and *M. tuberculosis* gyrase, and it is less pronounced for the flat CTD from *B. burgdorferi* (8,19,20). It has been proposed that the CTDs guide the DNA towards the gyrase core for strand passage (15,21). Recent single molecule (sm) FRET studies demonstrated that the CTDs are in a resting position close to the gyrase core in the absence of DNA, and they swing into an extended position when contacted by DNA protruding from the DNA-gate (27).

The GyrA-box, a highly conserved motif on the GyrA CTD, has been identified as a hallmark feature of gyrases that is not shared by topoisomerase IV that catalyse DNA decatenation (28), and it has been suggested to confer functional differences to these enzymes (5). The GyrA-box consists of the highly basic seven amino acid consensus sequence Q(R/K)RGG(R/K)G and is located in a peripheral loop on blade 1 that connects this segment to blade 6, resulting in ring closure (Figure 1B) (28). The loop harbouring the GyrA-box is not resolved in the *E. coli* and *M. tuberculosis* CTD structures (8,19), but is clearly resolved in the *X. campestris* CTD structure (5). The GyrA-box of the *B. burgdorferi* GyrA that deviates more strongly from the consensus sequence has also been resolved and shows more extensive contacts to blade 6 than in *X. campestris* GyrA (20). Similar to the deletion of the CTDs, deletion of the entire GyrA-box, or replacement by a stretch of alanines, abolishes DNA wrapping and DNA supercoiling, but does not affect DNA relaxation (29). It has been suggested that the GyrA-box directly binds to DNA extending from the DNA-gate, contributes to bending of this DNA (29) and directs it to the CTDs (8,29). Therefore, it is believed to ensure presentation of an adjacent T-segment, and thus inter-molecular strand passage, favouring supercoiling instead of decatenation (8).

Here, we have dissected the effect of the GyrA-box in *Bacillus subtilis* gyrase on individual steps in DNA supercoiling. We show that the GyrA-box is not required for DNA binding to the CTDs, but it affects the geometry of the bound DNA. A gyrase mutant that lacks the GyrA-box cannot adopt a conformation with a narrowed N-gate in response to DNA binding, and it shows uncoupling between DNA binding and ATP hydrolysis. Our data implicate the GyrA-box in bending the DNA bound to the CTDs, and in inter-domain communication that couples G-segment binding to T-segment presentation and capture, and DNA binding to the nucleotide cycle.

## MATERIALS AND METHODS

### Deletion and mutation of the GyrA-box

The GyrA-box (amino acids 527–533) was mutated to a seven-alanine stretch or deleted by site-directed mutagenesis, using the Quikchange protocol (Stratagene), with the *B. subtilis gyrA* gene, the gene coding for the GyrBA fusion protein, or the region coding for the GyrA CTD as a template. The primers sequences were GyrA_AboxA_for: 5′-GCATC AACTT ACCGC AGTGC AGCAG CGGCC GCAGC AGCTG TACAA GGTAT GGG-3′, GyrA_AboxA_rev 5′-CCCAT ACCTT GTACA GCTGC TGCGG CCGCT GCTGC ACTGC GGTAA GTTGA TGC-3′, GyrA_ΔAbox_for: 5′-CGTCT TCCTG CATCA ACTTA CCGCA GTGTA CAAGG TATGG GAACA AACG-3′ and GyrA_ΔAbox_rev: 5′-CGTTT GTTCC CATAC CTTGT ACACT GCGGT AAGTT GATGC AGGAA GACG-3′. Correct sequences were confirmed.

### Protein purification

*Bacillus subtilis* GyrA, GyrB, the GyrB–GyrA fusion (GyrBA) and heterodimeric GyrA were purified from *E. coli* as described before (17,27,30). The GyrA CTD (amino acids 500–808) was produced with an N-terminal hexa-histidine tag, followed by a TEV protease cleavage site, in *E. coli* Rosetta cells at 37°C for 24 h using auto-induction medium (31). The construct lacks the acidic C-terminal tail (amino acids 809–821) that has been previously shown to interfere with DNA wrapping by the isolated CTD and in GyrA, but not in context of gyrase (32). Cells were harvested by centrifugation, resuspended in 50 mM Tris–HCl pH 7.5, 1 M NaCl, 10 mM MgCl_2_ and 2 mM β-mercaptoethanol (buffer A) and disrupted in a microfluidizer. The crude extract was cleared by centrifugation, the supernatant adjusted to 20 mM imidazole, and passed over a Ni^2+^-NTA column. Proteins were eluted with buffer A containing 500 mM imidazole and were dialyzed twice against buffer A containing 300 mM NaCl and ∼1.0 µM TEV protease. Remaining fusion protein and the cleaved tag were removed using a second chromatography on Ni^2+^-NTA. The flowthrough containing the CTD was further purified using size-exclusion chromatography on a calibrated 16/60 Superdex 200-pg column equilibrated with buffer A containing 300 mM NaCl. The elution volume of the CTD was consistent with a monomer. Fractions containing the CTD were concentrated to 700–1200 μM, flash-frozen in liquid nitrogen and stored at −20°C.

### DNA substrates and purification

Forward and reverse strands constituting a 40-bp DNA wrapping substrate (20) and a 60-bp gate-DNA (14) were ordered from Purimex (Staufen, Germany). For fluorescence measurements, forward and reverse strands for the 40-bp DNA carried AlexaFluor 488 (A488) and AlexaFluor 546 (A546) at their 5′-ends, respectively. The 60-bp gate-DNA was internally labelled with A546 (27). Complementary strands were annealed by incubation at 95°C for 5 min and then cooling to 20°C in 30 min using a thermocycler.

Negatively supercoiled pUC18 plasmid was purified from *E. coli* XL1-Blue cells, using the Qiagen Midi prep kit. Relaxed pUC18 plasmid was produced from negatively supercoiled plasmid using *B. subtilis* gyrase as described previously (17). All plasmids were ethanol precipitated at the end of the purification.

### Steady-state ATP hydrolysis

ATP hydrolysis by GyrBA was monitored photometrically in a coupled enzymatic assay as described previously (14,17) in 50 mM Tris–HCl pH 7.5, 100 mM KCl and 10 mM MgCl_2_ (buffer B), supplemented with 0.2 mM NADH, 0.4 mM phosphoenolpyruvate and varying concentrations of ATP. GyrBA concentrations were 50 nM in the presence and 200 nM in the absence of 100 nM of negatively supercoiled pUC18. Data were analysed using the Michaelis–Menten equation (Equation 1). (1)
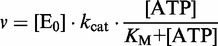
 where [E_0_] and [ATP] are the enzyme and ATP concentrations, and *v*, *k*_cat_ and *K*_M_ denominate the observed reaction velocity, the turnover number and the Michaelis–Menten constant, respectively.

### Topoisomerase activity

DNA relaxation by gyrase (200 nM GyrA, 800 nM GyrB, if not indicated otherwise) and GyrBA (200 nM) was monitored in 50 mM Tris–HCl pH 7.5, 100 mM KCl and 10 mM MgCl_2_, with 15 nM negatively supercoiled pUC18 plasmid as a substrate. DNA supercoiling was monitored with relaxed plasmid as a substrate in the presence of 2 mM ATP. Reactions were performed at 37°C for 2 min (supercoiling) or 30 min (relaxation), stopped by addition of 10 mM ethylenediaminetetraacetic acid, 1% (v/v) sodium dodecyl sulphate, 10% glycerol, 0.01% (w/v) bromphenol blue, and topoisomers were separated by electrophoresis on a 1.2% agarose gel as described in (14).

### Fluorescence anisotropy titrations

*K*_d_ values of GyrA–DNA complexes were determined in fluorescence anisotropy titration of 10 nM 60-bp gate-DNA carrying an internal A546 fluorophore as a probe (λ_ex_ = 555 nm, λ_em_ = 570 nm), with GyrA in the presence of 4 μM GyrB. Displacement with negatively supercoiled pUC18 was performed by titration of a preformed protein/DNA complex (10 nM DNA, 1 μM GyrA). Measurements were performed in buffer B at 37°C on a Horiba Jobin Yvon Fluoromax-3 Fluorimeter. *K*_d_ values of CTD-DNA complexes were determined in fluorescence anisotropy titrations of 100 nM 40-bp DNA labelled with A488 (λ_ex_ = 493 nm, λ_em_ = 517 nm) in 50 mM Tris–HCl, 40 mM KCl, 10 mM MgCl_2 _at 25°C. Data were evaluated according to Equation 2 as described (14): (2)
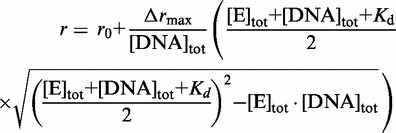

*r* is the apparent anisotropy, *r*_0_ and Δr_max_ the initial anisotropy and the maximal amplitude. [DNA]_tot_ and [E]_tot_ signify the total DNA and enzyme concentrations and *K*_d_ the dissociation constant.

To determine the influence of NaCl on DNA binding to the CTD, the fluorescence of 100 nM 40-bp DNA carrying A488 and A546 at the ends was monitored in the presence of 50 μM CTD at varying concentrations of NaCl, using the A546 fluorescence as a probe (λ_ex_ = 555 nm, λ_em_ = 570 nm). Under these conditions, A488 fluorescence was not excited.

### DNA bending

Bending of DNA by the CTD was measured by FRET using 100 nM A488/A546-labelled 40-bp DNA and 50 μM CTD in 50 mM Tris–HCl pH 7.5, 10 mM MgCl_2_ and varying concentrations of NaCl at 25°C. A488-fluorescence was excited at 460 nm to minimize acceptor excitation, and fluorescence emission spectra were recorded between 480 and 700 nm. The spectra were normalized by the sum of the fluorescence intensities at 518 and 571 nm (I_D_ and I_A_). An apparent FRET efficiency (E_FRET, app_) was calculated from the donor and acceptor fluorescence intensities at 518 (I_D_) and 571 nm (I_A_), respectively (Equation 3). (3)
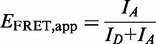



### smFRET experiments

GyrBA cysteine mutants or heterodimeric GyrA (30 μM) were labelled with 90 μM A488- (donor) and 120 μM A546- maleimide (acceptor) in buffer A containing 300 mM NaCl and 1 mM Tris(2-carboxyethyl)phosphine instead of β-mercaptoethanol at 25°C for 1 h. Excess dye was removed using size-exclusion chromatography with illustra Microspin G-25 columns (GE Healthcare). smFRET experiments were performed on a home-built confocal microscope at 37°C in buffer B as described previously (14,17), using 50 pM (donor concentration) fluorescently labelled GyrA or GyrBA, and 8 μM GyrB, 20 nM pUC18, 500 nM 60-bp gate-DNA and 2 mM 5′-adenosyl-β,γ-imidodiohosphate (ADPNP) (Sigma-Aldrich, St. Louis, USA or Jena Bioscience GmBH, Jena, Germany), if present.

Data analysis were restricted to fluorescence events with >100 photons. Donor and acceptor fluorescence intensities were corrected for background, and for cross-detection of photons, different detection efficiencies and quantum yields and direct acceptor excitation. Correction parameters for the heterodimeric GyrA/GyrA_T140C/K594C and GyrBA_S7C have been determined previously (17,27). A detailed description of the data analysis is given in (33).

### Differential scanning calorimetry (DSC)

The CTD was dialyzed against 50 mM potassium phosphate pH 7.5, 200 mM KCl, 10 mM MgCl_2,_ and diluted to 35–72 µM. Measurements were performed on a 6100 Nano Differential scanning microcalorimeter (Calorimetry Science Corp) with a temperature ramp from 20°C–90°C at a heating rate of 2°C/min. Heat capacity data were corrected for buffer contributions and different heat responses of the sample and reference cells and were analysed with a two-state model to obtain the midpoint temperature of unfolding and the unfolding enthalpy ΔH.

## RESULTS

### Deletion or substitution of the GyrA-box in *B. subtilis* gyrase abolishes DNA supercoiling

The GyrA-box, a seven amino acid motif in the CTD of *E. coli* GyrA, was shown to be a key determinant for DNA supercoiling (29). Based on sequence alignments (28), the GyrA-box in *B. subtilis* GyrA can be identified as the sequence ^527^QKRGGKG^533^. According to a homology model of the *B. subtilis* CTD, created with SWISS-MODEL (34) using the GyrA CTD from *X. campestris* (5) as a template, the GyrA-box is part of an extended loop connecting blades 1 and 6 of the *B. subtilis* CTD (Figure 1A and B). We first tested whether mutation or deletion of the GyrA-box affects the ATP-dependent DNA supercoiling and nucleotide-independent DNA relaxation activities of *B. subtilis* gyrase. To this end, we mutated all residues to alanines or deleted the entire GyrA-box (amino acids 527–533). Both mutants completely lost the capacity for ATP-dependent DNA-supercoiling activity, but they rapidly relax negatively supercoiled DNA (Figure 1C). Gyrase lacking the GyrA-box also showed increased relaxation in the presence of ATP and efficient decatenation activity (Supplementary Figure S1). Thus, the GyrA-box plays an important role in DNA supercoiling, but is dispensable for DNA relaxation and decatenation.

### The GyrA-box marginally stabilizes the CTD

**Figure 2.**
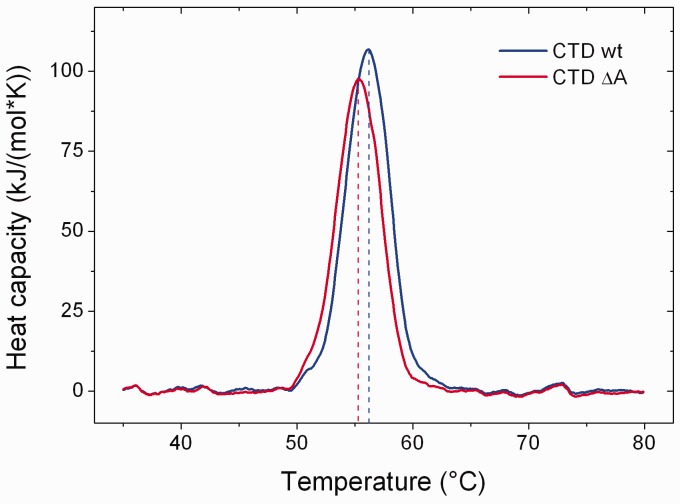
Thermodynamic stability of GyrA CTDs. Excess molar heat capacity plots for thermal unfolding of the wild-type CTD (blue) and the CTD lacking the GyrA-box (ΔA, red), corrected for buffer contributions. Transition midpoints are marked by dashed lines.

In a first step towards understanding the role of the GyrA-box for DNA supercoiling, we investigated its effect on the stability and folding of the isolated CTD in differential scanning calorimetry experiments (Figure 2). The wild-type CTD shows a symmetric, bell-shaped DSC profile on temperature-induced unfolding, indicating a one-step unfolding process with a transition temperature of 56.2 ± 0.2°C. The CTD lacking the GyrA-box unfolds with a slightly, but reproducibly lower midpoint of 55.3 ± 0.0°C. Unfolding of both proteins is not reversible, precluding a rigorous thermodynamic analysis of the data. Nevertheless, it is possible to calculate the enthalpy change associated with the unfolding event, which is 526 ± 18 kJ/mol for wild-type CTD, and 466 ± 13 kJ/mol for the CTD lacking the GyrA-box. Although these values have to be interpreted with care, they are in agreement with a small stabilizing effect of the GyrA-box on the CTD.

### Influence of the GyrA-box on DNA binding to the CTD and on the conformation of the bound DNA

**Figure 3.**
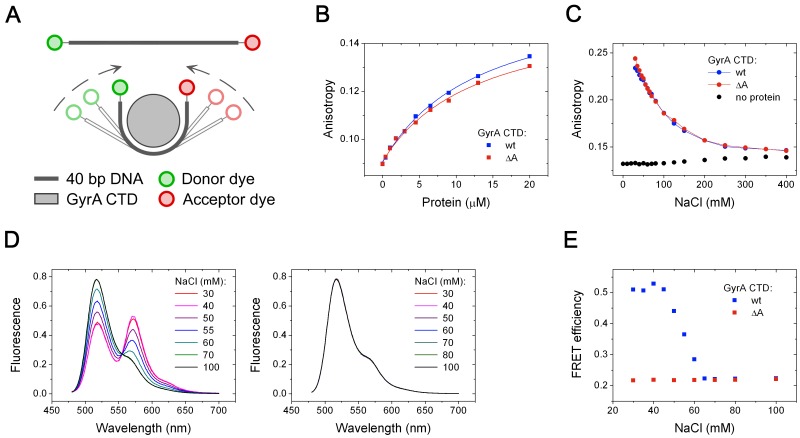
DNA binding and bending by GyrA CTDs. (**A**) Cartoon of the 40-bp DNA 5′-labelled with A488 (donor) and A546 dyes (acceptor). Binding and bending of the 40-bp DNA by the GyrA CTD results in a reduced end-to-end distance in the DNA and thus a decreased inter-dye distance that is detectable by FRET. (**B**) Fluorescence anisotropy titration of the A488-labelled 40-bp DNA with wild-type CTD (blue) and CTD lacking the GyrA-box (red). (**C**) Fluorescence anisotropy of the double-labelled 40-bp DNA [see (A)] in presence of 50 µM wild-type CTD (blue) and CTD lacking the GyrA-box (red) as a function of the NaCl concentration. The black points depict the anisotropy of the 40-bp DNA in the absence of proteins. (**D**) Fluorescence spectra of the double-labelled 40-bp DNA [see (A)] in presence of 50 µM wild-type CTD (left) and CTD lacking the GyrA-box (right) as a function of the NaCl concentration (30–100 mM). Under these conditions, ∼80% of the DNA is bound to the CTD. Spectra are normalized as described in ‘Materials and Methods’ section. (**E**) FRET efficiency as a function of the NaCl concentration, derived from the fluorescence spectra of the double-labelled 40-bp DNA in (D).

It has been shown previously that the CTDs bind DNA around their perimeter, leading to the introduction of writhe into the bound DNA (15,16,20). The GyrA-box contains a number of positively charged amino acid residues, suggesting a possible contribution of the GyrA-box to DNA binding. Therefore, we determined the dissociation constants of CTD/DNA complexes, using a 40-bp DNA (Figure 3 A and B). Because of the moderate DNA affinity of the CTD, the NaCl concentration had to be reduced to 40 mM in these experiments. The *K*_d_ values were virtually identical with 13.2 ± 1.4 μM for the wild-type CTD, and 13.1 ± 1.9 μM for the CTD lacking the GyrA-box. Thus, the GyrA-box has no effect on DNA binding to the gyrase CTD under these conditions.

To test whether the geometry of the DNA bound to the CTD is affected by the GyrA-box, we monitored bending of the 40-bp DNA substrate by the CTDs using FRET between donor (A488) and acceptor (A546) fluorophores attached to the ends of the DNA (Figure 3 A and D). At NaCl concentrations <40 mM, binding of the CTD to the DNA caused an increase in acceptor fluorescence because of FRET, consistent with bending of the DNA that is bound and wrapped around the CTD. The apparent FRET efficiency decreases with increasing NaCl concentrations, and reaches a constant (low) level at NaCl concentrations >60 mM (Figure 3D and E). In the presence of the CTD lacking the GyrA-box, in contrast, the acceptor fluorescence signal is not increased, but the apparent FRET efficiency remains at a low level corresponding to the low-FRET state of the DNA bound to the wild-type CTD at higher NaCl concentrations (Figure 3 D and E).

To confirm that the DNA is bound under the conditions where no FRET increase is observed, we measured the fluorescence anisotropy of A546 in the double-labelled DNA in the presence of the CTD as a function of the NaCl concentration (from 30 to 400 mM, Figure 3C). At low NaCl concentrations, the anisotropy is high and virtually identical in the presence of wild-type CTD and CTD lacking the GyrA-box. The anisotropy decreases with increasing NaCl concentration, but remains significantly higher than the anisotropy for the free DNA, confirming that the DNA in the FRET experiments is bound to the CTD at NaCl concentrations <60 mM. Thus, the differences in FRET reflect differences in the conformation of the DNA bound to the wild-type CTD and to the CTD lacking the GyrA-box.

Altogether, these results demonstrate that the GyrA-box does not significantly contribute to DNA binding to the CTD, but contributes to DNA bending and wrapping around the CTD and influences the geometry of the bound DNA. The influence of the GyrA-box on the DNA geometry may be an indirect effect because of the stabilization of the CTD and thus the DNA-binding surface by the GyrA-box.

### DNA-induced CTD movement is retained in gyrase lacking the GyrA-box

**Figure 4.**
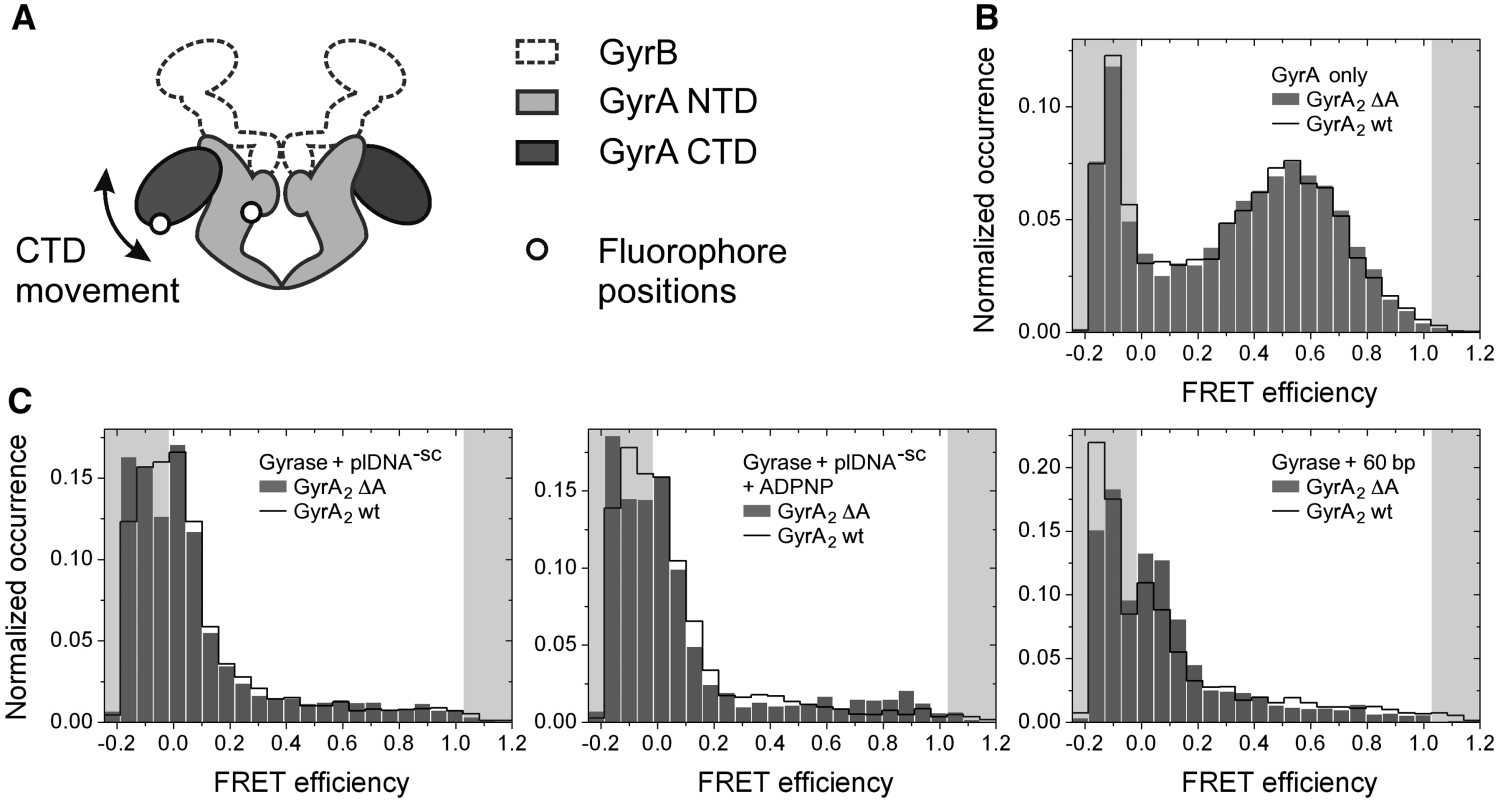
Movement of the GyrA CTD in response to DNA and nucleotide binding. (**A**) Cartoon depicting gyrase containing a GyrA heterodimer with one wild-type and one mutant subunit with cysteine residues introduced on the GyrA NTD and on the CTD for fluorescence labelling. (B and C) smFRET histograms for heterodimeric GyrA labelled with donor and acceptor dyes on one GyrA NTD and the corresponding CTD. Data for the wild-type enzyme and GyrA lacking the GyrA-box are depicted with a black line and grey bars, respectively. (**B**) smFRET histograms for GyrA in absence of DNA and nucleotides. (**C**) smFRET histograms for gyrase in presence of negatively supercoiled plasmid (left), with ADPNP (middle), in presence of a 60-bp DNA (right).

Deletion of the GyrA-box results in a slight structural destabilization of the CTD and alters the geometry of bound DNA, possibly affecting the overall wrapping of DNA around gyrase. We have previously shown that contacts of DNA substrates extending from the DNA-gate in gyrase with the CTDs initiate a movement of the CTDs, up and away from the gyrase body (27). To delineate the role of the GyrA-box in the DNA supercoiling reaction, we therefore tested the effect of the GyrA-box on this DNA-induced conformational change of gyrase. Heterodimeric GyrA containing one wild-type subunit and one subunit with donor and acceptor dyes close to the DNA-gate (T140C) and on segment 2 of the CTD (K594C) was used to monitor distance changes between the gyrase body and the CTDs in smFRET experiments (Figure 4A). Introduction of cysteines and fluorophores did not affect the DNA relaxation and supercoiling activities of gyrase (Supplementary Figure S2). Donor/acceptor-labelled GyrA exhibits a broad, but well-defined FRET histogram, with a maximum at E_FRET_=0.55, corresponding to the CTDs close to the gyrase core, as reported previously [Figure 4B, (27)]. A similar FRET histogram was obtained for GyrA lacking the GyrA-box (Figure 4B).

For gyrase containing the GyrA-box, addition of a 60-bp gate-DNA or of negatively supercoiled plasmid resulted in a FRET histogram with the main species of FRET = 0 [Figure 4C, (27)]. The decrease in E_FRET_ results from a movement of the CTDs away from the gyrase body that is induced by DNA sufficiently long to bridge the DNA-gate and the CTDs (27). Gyrase lacking the GyrA-box also displayed low-FRET efficiencies in the presence of these DNAs (Figure 4C), demonstrating that the DNA-induced movement of the CTDs does not require the GyrA-box. Subsequent addition of ADPNP to the gyrase/plasmid complex does not result in a change of the FRET efficiency (Figure 4C) (27), neither in the presence nor in the absence of the GyrA-box, indicating that the CTDs remain in the upwards position when the N-gate closes.

The similar response of the CTDs to DNA-binding suggests a similar binding mode of the DNA extending from the DNA-gate to the CTDs in both proteins. To test this hypothesis, we determined the effect of the GyrA-box on binding the 60-bp DNA (Supplementary Figure S3). *K*_d_ values were 63 ± 5 nM for wild-type gyrase and 50 ± 2 nM for gyrase lacking the GyrA-box indicating similar, although not identical, DNA affinities. Displacement titrations of the DNA/gyrase complex with (negatively supercoiled) pUC18 plasmid gave qualitatively similar results (Supplementary Figure S3), confirming that the GyrA-box does not contribute to affinity for the DNA extending from the DNA-gate to the CTDs.

Altogether, the GyrA-box is not required for the displacement of the CTDs induced by first contacts of the DNA flanking the gate-segment with the CTDs.

### Deleting the GyrA-box alters the behaviour of the N-gate

**Figure 5.**
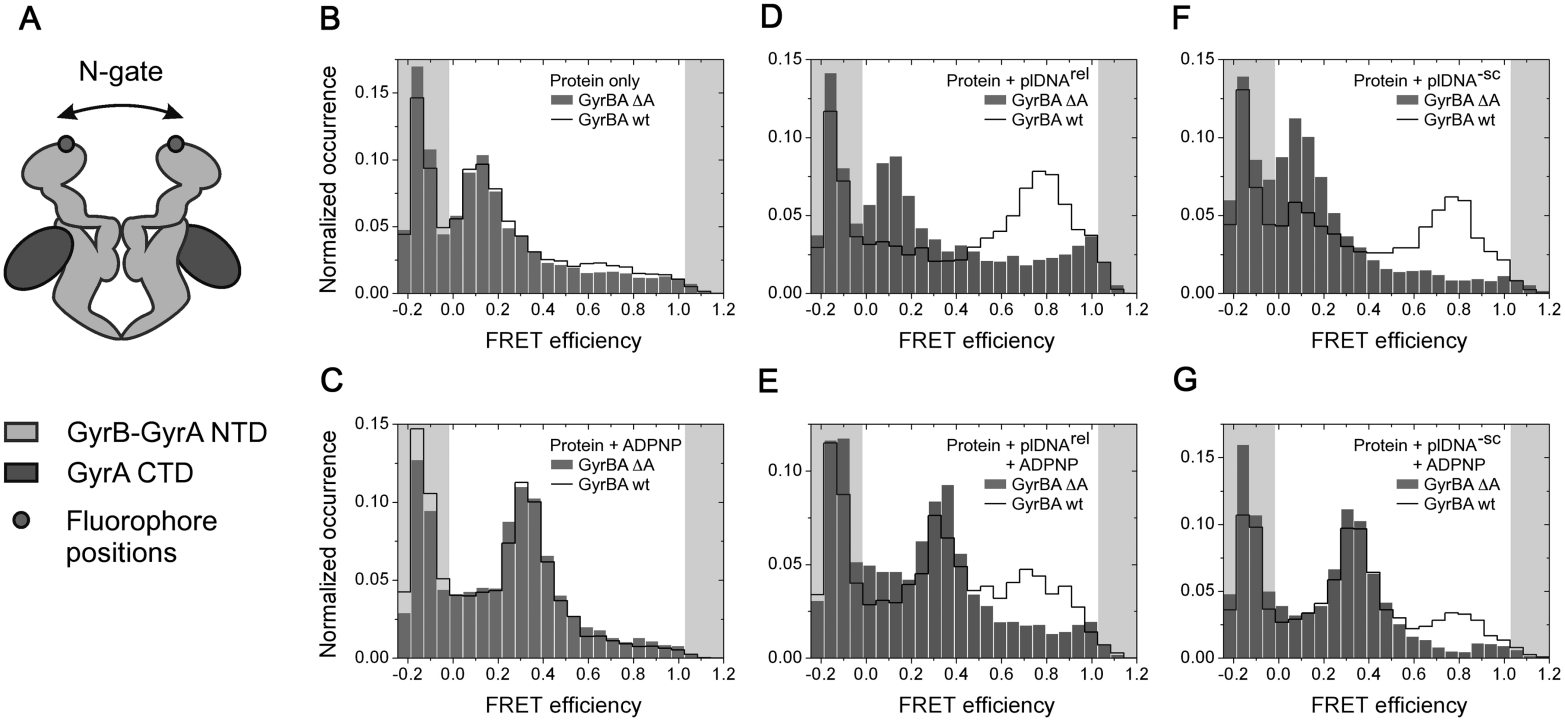
Conformational change in the gyrase N-gate induced by DNA and nucleotide binding. (**A**) Cartoon depicting gyrase formed by a dimeric GyrB-GyrA-fusion protein (GyrBA) containing cysteine residues at the N-gate for fluorescence labelling. (**B–G**) smFRET histograms for GyrBA labelled with donor and acceptor dyes at the N-gate. Data for the wild-type enzyme and GyrBA lacking the GyrA-box are depicted with a black line and grey bars, respectively. smFRET histograms for GyrBA (B), for GyrBA in the presence of ADPNP (C), in the presence of relaxed (D) and negatively supercoiled plasmid (F), and in the presence of relaxed (E) and negatively supercoiled plasmid (G) plus ADPNP.

The difference in DNA bending between the isolated wild-type CTD and the CTD lacking the GyrA-box may lead to different relative positions of the incoming DNA segment extending from the DNA-gate to the CTD, and the outgoing DNA segment delivered from the CTD back to gyrase. In other words, the relative position of the G- and T-segments could be affected by the GyrA-box. In a previous study, we showed that DNAs sufficiently long to wrap completely around the CTD perimeter cause a narrowing of the gyrase N-gate, thereby preparing T-segment capture by subsequent nucleotide-induced N-gate closure (17). To dissect the role of the GyrA-box in this conformational change, we performed smFRET experiments with a GyrBA fusion protein donor/acceptor-labelled at the N-gate [GyrBA_S7C, (17), Figure 5A]. GyrBA and fluorescently labelled GyrBA_S7C exhibit ATP-dependent plasmid supercoiling and nucleotide-independent plasmid relaxation activities similar to wild-type gyrase (17). GyrBA lacking the GyrA-box displays no ATP-dependent DNA supercoiling and slightly enhanced nucleotide-independent DNA relaxation activities (Supplementary Figure S4). Thus, the GyrBA fusion protein also recapitulates the behaviour of heterotetrameric gyrase with respect to the GyrA-box.

FRET histograms of donor/acceptor-labelled GyrBA_S7C with and without GyrA-box show mean FRET efficiencies of 0.15, indicative of an open N-gate [Figure 5B, (17)]. In the presence of the non-hydrolyzable ATP analogue ADPNP, the FRET efficiencies increase to 0.35 for both proteins (Figure 5C), corresponding to the closed conformation of the N-gate (i.e. dimerized ATP-binding domains in GyrB). Thus, the conformation of the N-gate in the absence and presence of ADPNP is not affected by the GyrA-box.

As reported previously, binding of relaxed plasmid (i.e. the substrate of the supercoiling reaction) leads to a FRET histogram with a mean FRET efficiency of 0.8, which we have been able to assign to a narrowed, but not yet closed N-gate [Figure 5D, (17)]. The FRET efficiency for the narrowed gate is higher than for the closed state because of the attachment site (17). In contrast to wild-type gyrase, when we performed the same experiment with GyrBA lacking the GyrA-box, the FRET efficiency remained at 0.15, indicating that the N-gate remains in the open conformation when plasmid binds. Similar behaviour was observed with negatively supercoiled plasmid (Figure 5F). Thus, these results implicate the GyrA-box in coupling of DNA binding and wrapping to N-gate narrowing.

Addition of ADPNP to a complex of GyrBA and relaxed plasmid reduces the high-FRET population (narrowed N-gate), and a medium-FRET population at E_FRET_ = 0.35 (closed N-gate) appears, indicating closure of the N-gate on ADPNP binding in a fraction of the molecules [Figure 5E, (16)]. As reported previously (17), the population that closes the N-gate in response to ADPNP binding most likely reflects the fraction of GyrBA that lacks a T-segment between the two GyrB arms before ADPNP addition, whereas the population that cannot close the N-gate reflects the fraction of GyrBA with a T-segment between the GyrB arms as a result of wrapping of the DNA around gyrase in a positive node (17). This assignment, although tentative in the absence of a direct measure for the presence of a T-segment, is in agreement with the different fractions of molecules closing in response to ADPNP binding when relaxed or negatively supercoiled DNA is bound to gyrase (17), with the observation that the cavity between closed N- and DNA-gates may be too small to accommodate a double-stranded DNA (6,18), and with the reduced coupling of ADPNP binding to strand passage compared with ATP (26). Although limited support of the supercoiling reaction has been shown for *E. coli* gyrase, we detected no supercoiling with *B. subtilis* gyrase in the presence of ADPNP (Supplementary Figure S5), and ADPNP may not support strand passage. However, we have previously shown that ADPNP binds to GyrB (30), induces N-gate closure (17) and affects the conformation of DNA bound to the DNA-gate of gyrase (14). Thus, ADPNP seems to be an adequate nucleotide analogue to investigate events in the catalytic cycle of *B. subtilis* gyrase that precede strand passage.

In contrast to the observation with wild-type GyrBA, in the corresponding experiment with GyrBA lacking the GyrA-box, bound to relaxed plasmid, ADPNP binding leads to a shift of the FRET efficiency to 0.35, and thus to N-gate closure, for the complete gyrase population (Figure 5E), suggesting that no T-segment has been present between the GyrB arms.

Wrapping with a positive handedness can be achieved more easily with relaxed DNA than with negatively supercoiled DNA. We have previously shown that binding of negatively supercoiled DNA to GyrBA does not induce N-gate narrowing with the same efficiency, leaving a fraction of GyrBA with an open N-gate [E_FRET_ = 0.15, Figure 5F, (17)]. Addition of ADPNP depopulates the state with E_FRET_ = 0.15 (open N-gate) and reduces the population with E_FRET_ = 0.8 (narrow N-gate), leading to the appearance of a species with E_FRET_ = 0.35 [closed N-gate, Figure 5G, (17)]. The fraction of GyrBA with a narrowed N-gate that closes the N-gate on ADPNP binding is larger compared with the experiment with relaxed plasmid, possibly because of the smaller fraction of GyrBA with DNA wrapped in a positive node, and thus with a T-segment between the GyrB arms (17). Again, GyrBA lacking the GyrA-box showed different behaviour: First, the FRET histograms in the presence of negatively supercoiled DNA were indistinguishable from the ones obtained with relaxed plasmid (Figure 5D and F), indicating that gyrase lacking the GyrA-box is not sensitive to the supercoiling state of the bound DNA. Second, ADPNP addition converts all molecules to a medium-FRET state corresponding to a closed N-gate (Figure 5E and G), indicating that, as with relaxed plasmid, no T-segment is present in the upper cavity. This interpretation is supported by increased ATP-independent (Figure 1) and ATP-dependent relaxation activities (Supplementary Figure S1) and efficient decatenation activity by gyrase lacking the GyrA-box (Supplementary Figure S1). Overall, the GyrA-box thus contributes to the geometry of the DNA wrapped around the CTDs, to coupling DNA binding with N-gate narrowing, and to T-segment presentation and capture.

### DNA-stimulated ATP hydrolysis is hampered in the GyrA deletion mutant

**Figure 6.**
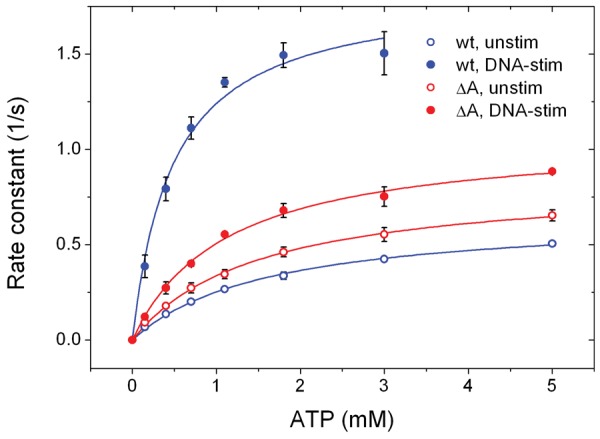
Steady-state ATPase activity of GyrB-GyrA-fusion proteins (GyrBA). Rate constants of ATP hydrolysis as a function of the ATP concentration for wild-type GyrBA (blue) and GyrBA lacking the GyrA-box (red) in absence (open circles) and presence of negatively supercoiled plasmid (filled circles).

The stimulation of the gyrase ATPase activity by DNA parallels the propensity of the DNA to induce narrowing of the N-gate (17). As N-gate narrowing does not occur in the absence of the GyrA-box, we finally tested if gyrase lacking the GyrA-box still exhibits DNA-stimulated ATPase activity (Figure 6 and Table 1). The *k*_cat_ values for ATP hydrolysis by GyrBA were 0.66 ± 0.02 s^−^^1^ in the absence and 1.84 ± 0.08 s^−^^1^ in the presence of negatively supercoiled plasmid, corresponding to a 2.8-fold stimulation by DNA. At the same time, the *K*_M,app_ values for ATP were reduced 3.4-fold from 1.61 ± 0.10 mM in the absence to 0.48 ± 0.07 mM in the presence of DNA (Figure 6), reflecting cooperative binding of DNA and ATP to GyrBA (17).

**Table 1.** Effect of the GyrA-box on DNA-stimulated ATPase activity

Protein	*k*_cat_ (s^−1^)	*K*_M,ATP _(mM)
GyrBA	0.66 ± 0.02	1.61 ± 0.10
GyrBA/DNA	1.84 ± 0.08	0.48 ± 0.07
GyrBA_ΔAbox	0.84 ± 0.02	1.49 ± 0.08
GyrBA_ΔAbox/DNA	1.07 ± 0.03	1.12 ± 0.08

GyrBA lacking the GyrA-box showed *k*_cat_ and *K*_M,app _values of 0.84 ± 0.02 s^−^^1^ and 1.49 ± 0.08 mM that were similar to wild-type (Figure 6), in-line with its wild-type-like response of the N-gate conformation to ADPNP (Figure 5C). In the presence of negatively supercoiled plasmid, the *k*_cat_ increased to 1.07 ± 0.03 s^−^^1^ (1.3-fold), and the *K*_M,app_ for ATP was reduced to 1.12 ± 0.08 mM (1.3-fold) (Figure 6). The deletion of the GyrA-box thus results in a severe uncoupling of DNA binding and ATP hydrolysis in gyrase, which is in agreement with the lack of N-gate narrowing in response to DNA binding.

Altogether, our results implicate the GyrA-box on the gyrase CTD in DNA bending, thereby determining the geometry of the incoming and outgoing DNA segments on the CTD. Furthermore, the GyrA-box mediates coupling of DNA binding on the CTD to N-gate conformation and coordinates DNA binding with ATP hydrolysis.

## DISCUSSION

The GyrA-box has been identified as a conserved element in the CTD of bacterial gyrases (28) that contributes to the introduction of writhe into DNA bound to the CTD and is required for DNA supercoiling, but not for DNA relaxation (29). We have dissected the role of the GyrA-box in *B. subtilis* gyrase in individual steps of the catalytic cycle. The GyrA-box does not contribute to DNA affinity of the CTDs, but to the geometry of the bound DNA. In the absence of the GyrA-box, DNA bound to gyrase does not induce a narrowing of the N-gate, and does not efficiently stimulate the gyrase ATPase activity, implicating the GyrA-box in inter-domain communication that couples DNA binding with the nucleotide cycle, and thus with strand passage and DNA supercoiling.

### The GyrA-box determines the geometry of DNA bound to the gyrase CTDs

The GyrA-box slightly stabilizes the CTD fold. The effect is small; however, it will diminish at physiological temperatures, suggesting rather subtle structural differences between wild-type CTD and a CTD lacking the GyrA-box. Several high-resolution structures of the GyrA CTD have been reported [*E. coli, B. burgdorferi*, *M. tuberculosis, X. campestris*, (5,8,19,20)]. Strikingly, the region containing the GyrA-box is not ordered in most of these CTDs. In the *X. campestris* CTD (5), polar contacts connect the first CTD segment to the C-terminal part of the sixth segment of the CTD, implying a contribution to the stability of the CTD β-pinwheel fold. Although highly positively charged, the GyrA-box does not contribute to the DNA affinity of the CTDs, but instead it affects the conformation of the DNA bound to the CTD. A CTD lacking the GyrA-box does not bend the bound DNA, in-line with the previous report of the GyrA-box in introducing writhe (29). The similar dissociation constants of the CTD/DNA complexes, but the different bending, suggest that the residues contributing to DNA binding are the same in the wild-type CTD and the CTD lacking the GyrA-box, but the interface is arranged differently, possibly because of an opening of the β-pinwheel. The extents of the differences in DNA geometry are difficult to assess. Corbett *et al.* (20) have estimated an inter-dye distance of 52 Å for a 40-bp DNA of the same sequence, bound to the CTD from *B. burgdorferi* gyrase, and deduced a bending angle of 180°, corresponding to a reversal of direction. The (apparent) FRET efficiency for the DNA bound to the CTD of *B. subtilis* gyrase points to a similar inter-dye distance of ∼54 Å, the Förster distance for the A488/A546-dye pair used, and suggests a similarly extreme bending of the DNA. The lack of detectable FRET for the same DNA bound to the CTD lacking the GyrA-box puts the inter-dye distance beyond 80 Å, consistent with less bending. DNA bending by the CTD is only observed at low-salt concentrations (<60 mM NaCl), although the CTD still binds DNA at higher ionic strengths. A similar sensitivity of bending to ionic strength has been described for the CTD from *E. coli* Topoisomerase IV and *B. burgdorferi* gyrase (20), suggesting that bending is the result of ionic interactions. The rather low-DNA affinities of the CTDs are consistent with deformation of the DNA and with high-dissociation rates. Apparently, DNA bending is not very stable and may only be transient.

### The GyrA-box contributes to inter-domain communication in gyrase

DNA binding and the nucleotide cycle of gyrase are coordinated through a set of ligand-induced conformational changes that ultimately couple ATP binding and hydrolysis to DNA supercoiling. DNA is bound at the DNA-gate of gyrase, and distorted (14). DNA regions flanking this gate-DNA extend from the gyrase body and establish first contacts with the CTDs, causing the CTDs to swing into an extended position away from the gyrase body (27). DNA sufficiently long to wrap around the perimeter of the CTD then induces a conformational change of the N-gate [narrowing, (17)] that prepares T-segment capture by nucleotide-induced N-gate closure. DNA-stimulated ATP hydrolysis is believed to occur when the N-gate is closed, and a T-segment is captured in the upper cavity (18). We have previously shown that the fraction of gyrase/DNA complexes with a narrowed N-gate correlates with the stimulation of the ATPase activity by the corresponding DNA (17), possibly because gyrase spends less time in slowly hydrolyzing states.

**Figure 7.**
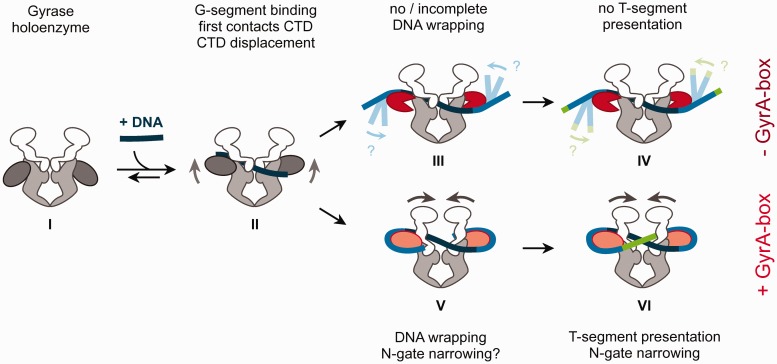
Model of DNA wrapping at the beginning of the supercoiling reaction and the role of the GyrA-box in DNA-induced conformational changes and T-segment presentation. DNA (dark blue) binds to the gyrase DNA-gate (I, II). Contacts of the flanking DNA regions with the CTDs cause a displacement of the CTDs away from the gyrase body (II). Binding of the DNA at the perimeter of the CTDs (light blue) leads to narrowing of the N-gate (V). The GyrA-box restricts the bound DNA in a conformation that leads to wrapping of the DNA around gyrase in a positive node, and to presentation of a T-segment (green) over the G-segment in the correct geometry for strand passage and negative supercoiling (VI). In the absence of the GyrA-box, DNA wrapping by the CTDs is incomplete (III), and the N-gate does not narrow in response to DNA binding. The lack of fixation of the DNA at the end of the wrap leads to a different geometry of the DNA bound to gyrase. The resulting different position of the T-segment is not suitable for strand passage towards DNA supercoiling (IV).

The CTDs of gyrase lacking the GyrA-box still undergo the DNA-induced upwards movement, indicating that the GyrA-box does not influence the early stages of DNA wrapping. However, the subsequent conformational change, N-gate narrowing induced by complete wrapping of DNA around the CTDs, is absent in gyrase lacking the GyrA-box (Figure 7). In agreement with the lack of DNA-induced N-gate narrowing, the ATPase activity of gyrase lacking the GyrA-box is not efficiently stimulated by DNA, although the GyrA-box is dispensable for nucleotide-induced N-gate closure. These results underline the importance of this conserved element in coordinating DNA binding and the nucleotide cycle. As a consequence of its role for DNA bending, the GyrA-box determines the relative arrangement of incoming and outgoing DNA segments from the CTD, and thus the relative position of the G- and T-segments of DNA bound to gyrase. The supercoiling deficiency of gyrase lacking the GyrA-box deletion can be attributed to the failure of bending DNA and of redirecting the DNA to the upper cavity of the enzymatic core, where it would serve as the T-segment in strand passage (Figure 7). Overall, our data are consistent with a role of the GyrA-box ‘at the end of the wrap’ that has been suggested earlier (21).

### Implications for the supercoiling cycle of DNA gyrase

Here, we have shown that the GyrA-box, in contrast to previous suggestions (8,29), does not direct the DNA from the DNA-gate to the CTDs, but it is a crucial element in directing the exiting DNA from the CTD towards GyrB and into the upper gyrase cavity. As a consequence, it orients the DNA before DNA supercoiling, possibly to ensure presentation of an adjacent T-segment and inter-molecular strand passage. At the same time, it contributes to the coordination of DNA binding with the N-gate conformation and ATP hydrolysis, leading to a coupling of DNA binding, ATP hydrolysis and DNA supercoiling. Together with our previous findings of DNA-induced conformational changes of the CTDs and the N-gate, it becomes clear that the conformational cycle of gyrase is not only driven by nucleotide binding and hydrolysis, but also tremendously affected by the bound DNA substrate. The highly inter-connected events at the beginning of the catalytic cycle cause a cascade of conformational changes that coordinate DNA binding with the nucleotide cycle and ultimately lead to strand passage and DNA supercoiling.

## SUPPLEMENTARY DATA

Supplementary Data are available at NAR Online: Supplementary Figures 1–5 and Supplementary Reference [13].

## FUNDING

VolkswagenStiftung and Swiss National Science Foundation. Funding for open access charge: University of Muenster.

*Conflict of interest statement*. None declared.

## Supplementary Material

Supplementary Data
