# Supplementary material for: The GyrA-box determines the geometry of DNA bound to gyrase and couples DNA binding to the nucleotide cycle
Source: Nucleic Acids Res. 2012 Sep 12;40(21):10893–903. doi: 10.1093/nar/gks852 (PMC3510516; doi:10.1093/nar/gks852)
Supplement: Supplementary Data [file supp_40_21_10893__index.html]

The GyrA-box determines the geometry of DNA bound to gyrase and couples DNA binding to the nucleotide cycle — The GyrA-box determines the geometry of DNA bound to gyrase and couples DNA binding to the nucleotide cycle — Supplementary Data 

# The GyrA-box determines the geometry of DNA bound to gyrase and couples DNA binding to the nucleotide cycle

## Supplementary Data

files

**Files in this Data Supplement:**

- Supplementary Data - pdf file
