# Supplementary material for: The GyrA-box determines the geometry of DNA bound to gyrase and couples DNA binding to the nucleotide cycle
Source: Nucleic Acids Res. 2012 Sep 12;40(21):10893–903. doi: 10.1093/nar/gks852 (PMC3510516; doi:10.1093/nar/gks852)
Supplement: Supplementary Data [file supp_gks852_nar-01421-f-2012-File009.pdf]

Figure S1:

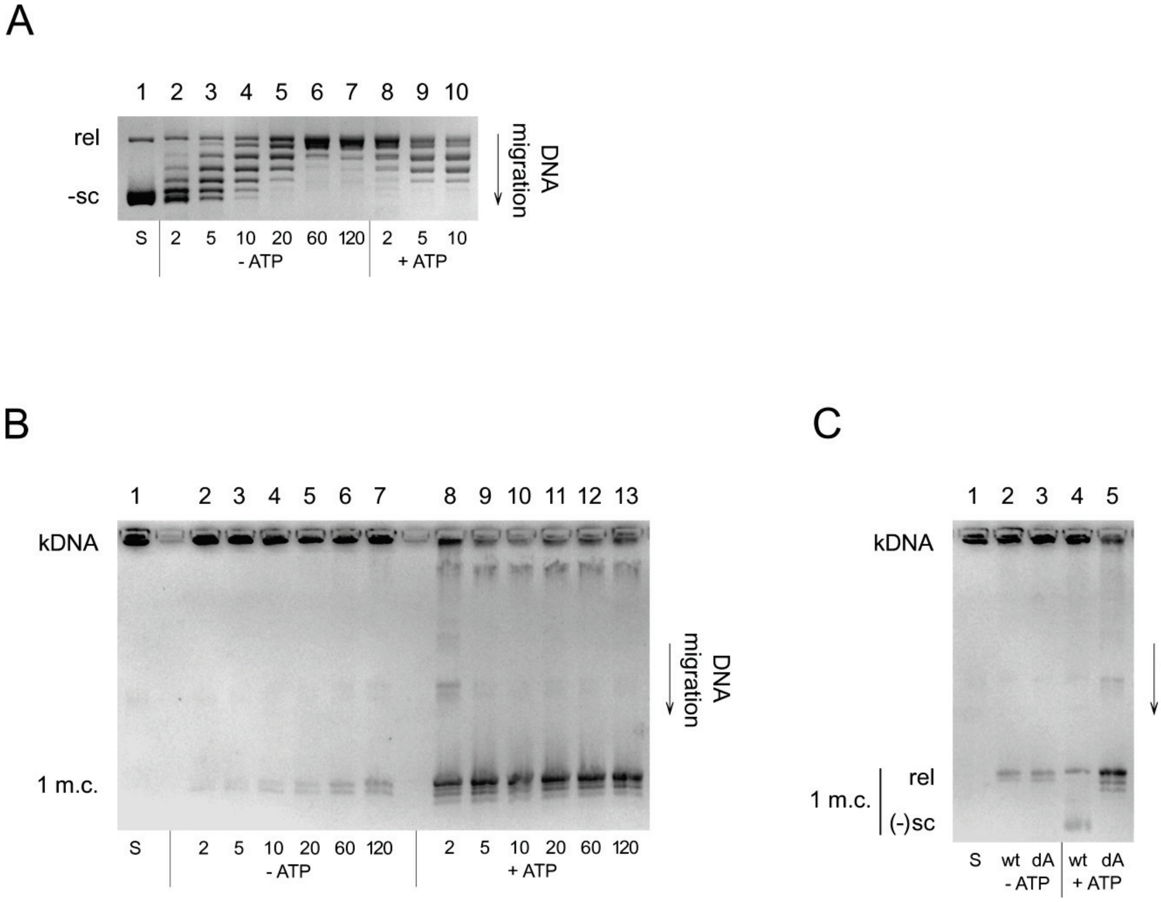

Figure S1: ATP-dependent relaxation and decatenation activities of *B. subtilis* gyrase and gyrase lacking the GyrA-box.

A) ATP-dependent DNA relaxation by gyrase. Gyrase lacking the GyrA-box relaxes negatively supercoiled DNA in the absence of ATP (lanes 2-7). The relaxation is more rapid in the presence of ATP (lanes 8-10). Lane 1 shows the negatively supercoiled DNA used as a substrate. Relaxation reactions were performed with 200 nM GyrA and 800 nM GyrB in 50 mM Tris/HCl pH 7.5, 100 mM KCl, and 10 mM $MgCl_2$ with 15 nM negatively supercoiled pUC18 plasmid as a substrate in the presence of 2 mM ATP at 37°C. At the indicated times (numbers below the gel, in minutes), the reaction was stopped by addition of 10 mM EDTA, 1% (v/v) SDS, 10% glycerol, 0.01% (w/v) bromphenol blue, and topoisomers were separated by electrophoresis on a 1.2% agarose gel as described (13).

B) Decatenation activity of gyrase and gyrase lacking the GyrA-box. Gyrase is not an efficient decatenase. In contrast, gyrase lacking the GyrA-box can decatenate kDNA (lane 1) inefficiently in the absence (lanes 2-7), and rapidly and efficiently in the presence of ATP

(lanes 8-13). The minicircles generated are marked with m.c. For decatenation reactions, 12.5 ng/µl kDNA (Inspiralis Ltd., UK, lane 1) was incubated with 100 nM GyrA and 400 nM GyrB in 50 mM Tris/HCl (pH 7.5), 100 mM KCl, 10 mM $MgCl_2$) in the absence and in the presence of 2 mM ATP at 37°C for the indicated times (denoted below the gel, in minutes). Reactions were stopped by addition of 10 mM EDTA, 1% (v/v) SDS, 10% glycerol, 0.01% (w/v) bromphenol blue, and topoisomers were separated by electrophoresis on a 1% agarose gel.

C) Comparison of decatenation activities of gyrase and gyrase lacking the GyrA-box (dA). Reactions were performed as in B), and stopped after 2 min (+ATP) and 60 min (-ATP). In the absence of ATP (lanes 1,2), gyrase with and without GyrA-box inefficiently decatenate kDNA (lane 1). In the presence of ATP, gyrase (lane 4) is an inefficient decatenase, as evidenced most clearly by the large fraction of remaining kDNA. The small fraction of minicircles generated is negatively supercoiled. In contrast, gyrase lacking the GyrA-box is more efficient in decatenation, as evidenced by the smaller fraction of kDNA remaining, and the minicircles generated are relaxed due to the lack of supercoiling activity.

Figure S2:

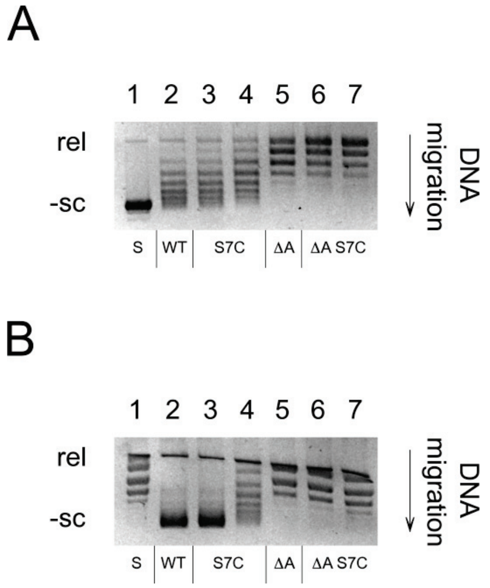

Figure S2: DNA relaxation and supercoiling by fluorescently labeled gyrase and gyrase lacking the GyrA-box.

Plasmid relaxation (lanes 2 – 6) and supercoiling (lanes 8 – 12) by GyrA wt (2, 8), GyrA_ΔA (3, 9), non-labeled GyrA_ΔA with cysteine residues introduced (4, 10), GyrA_ΔA with cysteines fully labeled with A488 (5, 11) and A546 (6, 12). Substrates for the relaxation and supercoiling reaction were negatively supercoiled (1) and relaxed plasmid (7), respectively. Nucleotide-independent DNA relaxation reactions were performed in 50 mM Tris/HCl pH 7.5, 100 mM KCl, and 10 mM $MgCl_2$ with 200 nM GyrA proteins, 800 nM GyrB, and 15 nM relaxed plasmid. ATP-dependent supercoiling reactions were performed in the same buffer using 50 nM GyrA proteins, 800 nM GyrB, 50 nM relaxed plasmid, and 2 mM ATP.

Figure S3:

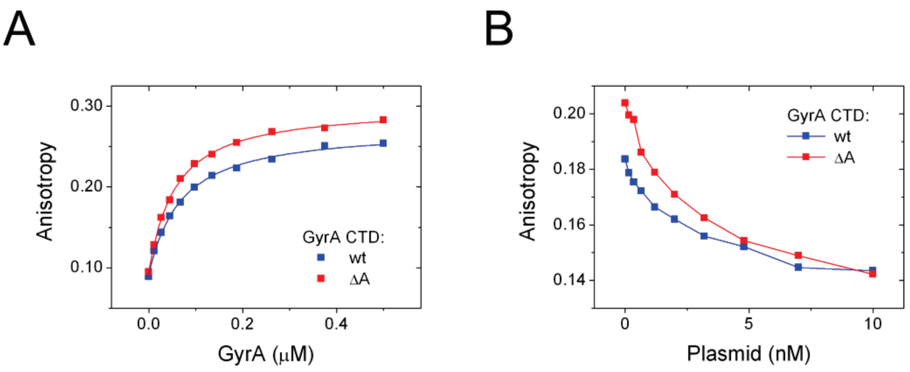

Figure S3: DNA binding by gyrase and gyrase lacking the GyrA-box monitored by fluorescence anisotropy.

A Titration of a 60 bp DNA labeled with A546 with GyrA (blue) and GyrA lacking the CTD (GyrA_ΔA, red) in 50 mM Tris/HCl pH 7.5, 100 mM KCl, and 10 mM $MgCl_2$ in the presence of GyrB. The anisotropy signal is plotted against the concentration of GyrA protein. Data were evaluated according to eq. 1 as described (1):

$$r = r_0 + \frac{\Delta r_{max}}{[DNA]_{tot}} \left(\frac{[E]_{tot} + [DNA]_{tot} + K_d}{2} \sqrt{\left(\frac{[E]_{tot} + [DNA]_{tot} + K_d}{2} \right)^2 - [E]_{tot} \cdot [DNA]_{tot}} \right) \qquad \text{eq. 1}$$

r is the apparent anisotropy, r_0 and Δr_{max} the initial anisotropy and the maximal amplitude. $[DNA]_{tot}$ and $[E]_{tot}$ signify the total DNA and enzyme concentrations, and K_d the dissociation constant. The resulting K_d values are 63 ± 5 nM (gyrase), and 50 ± 2 nM (gyrase lacking the GyrA-box).

B Displacement of a 60 bp DNA labeled with A546 by negatively supercoiled plasmid from GyrA (blue) and GyrA_ΔA (red) in the presence of GyrB. The lines connect the data points as a guide to the eye.

Figure S4:

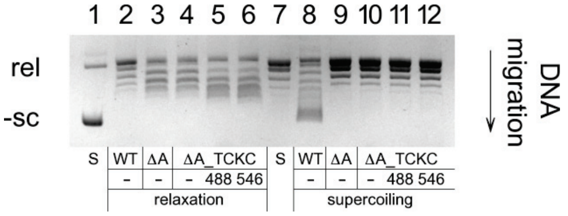

Figure S4: DNA relaxation and supercoiling reactions by fluorescently labeled GyrBA and GyrBA lacking the GyrA-box.

A: Nucleotide-independent plasmid relaxation by GyrBA (2), GyrBA with cysteines introduced at the N-gate (S7C; 3), GyrAB_S7C labeled with A488 and A546 (4), GyrBA lacking the GyrA-box (GyrBA_ΔA, 5), GyrBA_ΔA_S7C (6), GyrBA_ΔA_S7C labeled with A488 and A546 (7). Lane (1) shows negatively supercoiled plasmid, the substrate of the relaxation reaction. Reactions were performed as described in the legend to Figure S1.

B: ATP-dependent negative supercoiling of plasmid by GyrBA (2), GyrBA with cysteines introduced at the N-gate (S7C; 3), GyrAB_S7C labeled with A488 and A546 (4), GyrBA_ΔA (5), GyrBA_ΔA_S7C (6), GyrBA_ΔA_S7C labeled with A488 and A546 (7). Lane (1) shows relaxed plasmid, the substrate of the supercoiling reaction.

Figure S5:

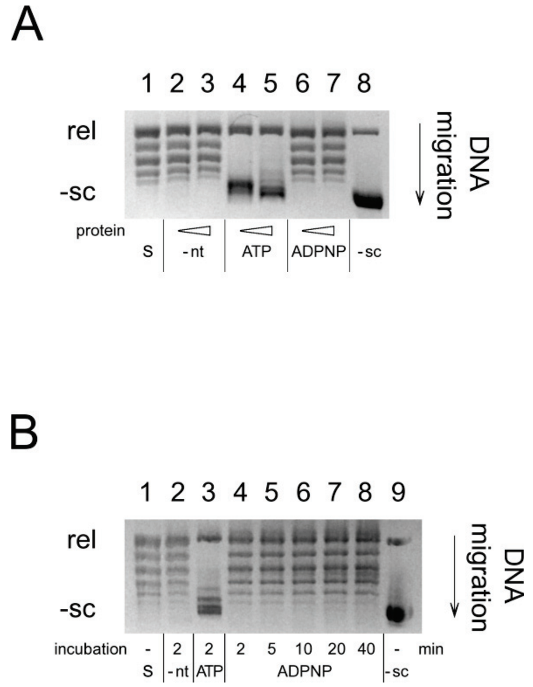

Figure S5: Supercoiling by *B. subtilis* gyrase: effect of nucleotides.

A: relaxed plasmid (rel, 1), supercoiling in the absence of nucleotide (2, 3), in the presence of ATP (4, 5), and in the presence of ADPNP (6, 7). Protein concentrations were 200 nM GyrA and 800 nM GyrB in lanes 2, 4, and 6, and 4 μM GyrA and 4 μM GyrB in lanes 3, 5, and 7. Negatively supercoiled plasmid (-sc, 8). Reaction times were 2 min.

B: (1) relaxed plasmid (rel, 1), supercoiling with 1.6 μM GyrA and 2.0 μM GyrB in the absence of nucleotide (2), in the presence of 2 mM ATP (3), and in the presence of 2 mM ADPNP (4-8). Incubation times in minutes are given below the gel. Negatively supercoiled plasmid (-sc, 9). Reactions were performed as described in the legend to Figure S1.

Reference:

1. Gubaev, A., Hilbert, M. and Klostermeier, D. (2009) The DNA Gate of *Bacillus subtilis* gyrase is predominantly in the closed conformation during the DNA supercoiling reaction. *Proc. Natl. Acad. Sci. U. S. A.*, **106**, 13278-13283.
